# Diarrhea Morbidities in Small Areas: Accounting for Non-Stationarity in Sociodemographic Impacts using Bayesian Spatially Varying Coefficient Modelling

**DOI:** 10.1038/s41598-017-10017-6

**Published:** 2017-08-30

**Authors:** F. B. Osei, A. Stein

**Affiliations:** 1 Department of Mathematics and Statistics, University of Energy and Natural Resources, Sunyani, Ghana; 2 Faculty of Geo-Information Science and Earth Observation (ITC), University of Twente, Enschede, Netherlands

## Abstract

Model-based estimation of diarrhea risk and understanding the dependency on sociodemographic factors is important for prioritizing interventions. It is unsuitable to calibrate regression model with a single set of coefficients, especially for large spatial domains. For this purpose, we developed a Bayesian hierarchical varying coefficient model to account for non-stationarity in the covariates. We used the integrated nested Laplace approximation for parameter estimation. Diarrhea morbidities in Ghana motivated our empirical study. Results indicated improvement regarding model fit and epidemiological benefits. The findings highlighted substantial spatial, temporal, and spatio-temporal heterogeneities in both diarrhea risk and the coefficients of the sociodemographic factors. Diarrhea risk in peri-urban and urban districts were 13.2% and 10.8% higher than rural districts, respectively. The varying coefficient model indicated further details, as the coefficients varied across districts. A unit increase in the proportion of inhabitants with unsafe liquid waste disposal was found to increase diarrhea risk by 11.5%, with higher percentages within the south-central parts through to the south-western parts. Districts with safe and unsafe drinking water sources unexpectedly had a similar risk, as were districts with safe and unsafe toilets. The findings show that site-specific interventions need to consider the varying effects of sociodemographic factors.

## Introduction

Disease indices, such as the relative risk, of common morbidities are important criteria for comparison of neighborhood health status, neighborhood health planning, and health budgetary allocations. With the changing population redistribution and deformation of sociodemographic characteristics, up to date statistic is required for planning. For diarrhea, reducing morbidity levels is closely linked to the attainment of the Millennium Development Goal 4 of reducing child mortality by two-thirds of the 1990 estimates. Globally, over 1.7 billion episodes are recorded every year with the majority of these occurring in low and middle-income countries^1–5^. Five important pathogens, rotavirus, enteropathogenic *E*. *coli*, enterotoxigenic *E*. *coli*, calicivirus, and *shigella* ^6, 7^ cause the majority of diarrhea cases. The pathogens can spread from feces through multiple exposure pathways (water, food, flies, soil, fingers, fomites) which have a complex web of interactions^8^. The past years have seen much developmental efforts on therapeutic treatments such as oral rehydration therapy, zinc, and nutrient supplementation, leading to dramatic reductions in mortalities^9, 10^. Morbidities, however, have declined only moderately^4^. Reduction of poverty and malnutrition, improving access to safe water and living conditions, provision of adequate sanitation and health care remain the formidable approach to reducing diarrhea in low and middle-income countries^8, 11^. The inadequacy of resources demands prioritization of relatively high risks areas during interventions. The often available national and regional level estimates result in a major setback to prioritization. Local estimates are therefore necessary. These should be unbiased and reliable to inform good practices. Controlling for sociodemographic effects could lead to precise risk estimates. Also, understanding diarrhea risk relationship with sociodemographic characteristics opens up the pathway for developing effective strategies to combat this menace.

Mapping diarrhea risk can provide a better understanding of the geographical variation of neighborhood health status. Ecological regression models have commonly been used to account for relevant confounding sociodemographic covariates to provide (1) reliable estimates of risk, and (2) estimates of the association between risk and covariates. When geographic information on neighborhoods is available, the inclusion of independent Gaussian and conditional autoregressive (CAR) processes as spatially varying intercepts could be adequate to account for residual spatial effects. These inclusions have the advantage to account for variance instabilities due to heterogeneous populations, unobserved influential factors, spatial interactions induced by similar sociodemographic conditions, and improve prediction accuracy. Wakefield^12^ provides discussions on disease mapping using ecological regression models. These, however, are fixed effect models and estimate a single coefficient for each covariate based on the implicit assumption of stationarity in the effect parameters. For most diseases, given the complexity of the relationship with sociodemographic factors, non-stationarity in the impacts of neighborhood sociodemographic factors is plausible^13^. For diarrhea, the assumption of stationarity in neighborhood sociodemographic effects is difficult to meet because of differences in neighborhood specific characteristic and unobserved factors that can locally influence disease outcomes. A mix of attributable socioeconomic inequalities such as low-income level, illiteracy, inadequate water and sanitation, urbanization^14–18^ are expected to exhibit spatially varied impacts. For instance, the effect of urbanization on diarrhea has two opposing facets; on the one hand, urbanization fueled by economic growth along with improvements in amenities such as availability safe water sources and good sanitation practices will reduce diarrhea morbidities. On the other hand, unplanned rapid urbanization fueled by rural-urban migration can enhance substantial increases in diarrhea morbidities due to stress on existing amenities which do not meet the demands of the rising population. If the neighborhood sociodemographic effects are allowed to vary by location, then we can remedy the preceding concerns.

The methodology in this paper is an alternative to the common ecological regression approach for disease mapping where covariates effects are assumed to be homogenous across neighborhoods. Non-stationarity impacts of the covariates may be accommodated by geographically weighted regression (GWR)^13^, or spatially varying coefficient (SVC) models^19^. GWR focuses on metric outcomes. Those, however, are less frequently encountered in disease mapping. The exception, however, is directly observed outcomes like incidence or prevalence rates. The robustness of the GWR relies on the selection of appropriate bandwidth for the kernel function which is dependent on the geographic locations of point reference data^20^. In this study, our interest lies in modeling the relative risk as an unobserved latent random variable. SVC models can easily be extended to latent random variable model and formulated within a hierarchical Bayesian framework. Gelfand *et al*.^19^ provide extensive theoretical discussions on SVC models using Gaussian random fields on point referenced data, and extensions to the spatio-temporal domain. There are still specification and implementation concerns for discrete disease data. Hence its application is rarely found in disease mapping literature. Morbidity data often consist of aggregates for administrative areas. For these reasons, we focus on modeling the spatially varying coefficients as realizations of Conditional Autoregressive Processes (CAR). Our empirical application is motivated by diarrhea epidemiology which remains as one of the top 5 out-patient morbidities in Ghana. The manuscript intends to demonstrate the methodological significance and the substantive epidemiological implications.

We adopt a hierarchical Bayesian modeling framework and consider a variety of hierarchical models that account for spatial random effects, temporal random effects, and spatio-temporal interaction effects. Markov Chain Monte Carlo (MCMC) simulations of numerically evaluating complex integrals can be time consuming and discouraging. We used Integrated Nested Laplace Approximation (INLA) for approximate Bayesian inference^21, 22^. The study has twofold objectives; (1) map area specific relative risk estimates, and (2) estimate the spatially varying association between relative risk and potential risk factors. In what follows, we present the model specifications and estimation methods, the data description and empirical applications. We finally end with the results and some discussions.

## Methods

We consider the double $\{{y}_{it},{n}_{it}\}$ representing the spatio-temporal outcomes of diarrhea and population data disaggregated by districts $i=1,\mathrm{...}m$ and temporal periods $t=1,\mathrm{...},T$. Such sampling models are typically realizations from the Poisson process ${Y}_{it} \sim {\rm{P}}({E}_{it}{\varsigma }_{it})$ with likelihood

1 $${y}_{it}|{\varsigma }_{it}=\prod _{it}\frac{{e}^{[{E}_{it}{\varsigma }_{it}]}{({E}_{it}{\varsigma }_{it})}^{{y}_{it}}}{{y}_{it}!}$$

where ${\varsigma }_{it}$ is the relative risk and ${E}_{it}$ is the expected number of cases. Of particular interest to policy makers and health strategists are inequalities in the relative risk ${\varsigma }_{it}$. The conventional estimate of area specific relative risk is the ratio of observed cases to population, ${\varsigma }_{it}={y}_{it}/{E}_{it}$, where the expected number of cases ${E}_{it}={r}_{t}{n}_{it}$ is defined, in the absence of covariates, as the number of cases defined in an epidemiologic “null model” of incidence. The risk variable ${r}_{t}$ is the individual level constant baseline risk estimated from the aggregated population via ${\hat{r}}_{t}={\sum }_{i=1}^{m}{y}_{it}/{\sum }_{i=1}^{m}{n}_{it}$. Population heterogeneity would influence variation in the relative risk estimates, and consequently, any estimated fixed effect parameter for areas of relatively small populations will show the highest variability^23^. Imposing independent normal prior distribution on the log-relative risk, $\mathrm{log}\,{\varsigma }_{it} \sim N(\alpha ,{\sigma }_{v}^{2})$, results to a log-linear regression with exchangeable random intercepts that corrects for population heterogeneity amongst areas. Thus $\mathrm{log}\,{\varsigma }_{it}=\alpha +{v}_{i}$, implying ${\varsigma }_{it}=\exp (\alpha +{v}_{i})$, where ${v}_{i} \sim N(0,{\sigma }_{v}^{2})$ and $p(v)\propto {\sigma }_{v}^{-n}\exp \{-0.5{\sigma }_{v}^{-2}{\rm{\Sigma }}{v}^{2}\}$. Here, *α* denotes the overall level of the relative risk on a log scale and ${v}_{i}$ denotes the exchangeable random intercepts across districts. It is conceivable that the relative risks over a set of contiguous areas are likely to demonstrate spatial correlation. Unmeasured confounders are potentially continuous in space and can exhibit spatial correlation. Such confounders are often accommodated by introducing a spatially correlated random effect term *u*_*i*_ which specifies the distribution of ${u}_{i}$ conditional on the set ${u}_{-i}={u}_{j\ne i}=\{{u}_{1},\mathrm{...},{u}_{i-1},{u}_{i+1},\mathrm{...},{u}_{n}\}$. A widely used scheme of representation under irregularly shaped areas is the intrinsic conditional autoregressive (ICAR) prior^24^ where the conditional distribution of ${u}_{i}$ is given by

2 $${u}_{i}|{u}_{-i=j} \sim N({\bar{u}}_{i},\frac{{\sigma }_{u}^{2}}{{w}_{i+}})$$

${\bar{u}}_{i}={\sum }_{j}{w}_{ij}{u}_{j}/{w}_{i+}$ and ${w}_{i+}={\sum }_{-i=j}{w}_{ij}$. In practice, ${w}_{ij}$ is an *n* × *n* weight matrix which captures the spatial proximity structure such that ${w}_{ij}=1$ if *i* and *j* are neighbors $i \sim j$(share a common boundary), and 0 otherwise. Setting ${w}_{ii}=0$ ensures that a location will not be used to predict itself. The above specification leads to the prior joint distribution

3 $$p({u}_{i}|{\sigma }_{u}^{2})\propto {\sigma }_{u}^{-n}\exp \{-0.5{\sigma }_{u}^{-2}{\sum }_{i=1}{\sum }_{j < i}{w}_{ij}{({u}_{i}-{u}_{j})}^{2}\}$$

This prior is not proper as it is based on paired differences. Imposing a sum to zero constraint $\sum {u}_{i}=0$ ensures identifiability. The mean of the joint prior distribution $p({u}_{i}|{\sigma }_{u}^{2})$ is therefore set to zero, where it’s precision matrix ${{\rm{\Sigma }}}_{u}$ has diagonal elements $\sum _{-i=j}{w}_{ij}{\sigma }_{u}^{-2}$ and off-diagonal elements $-{w}_{ij}{\sigma }_{u}^{2}$. For brevity, we write ${u}_{i} \sim ICAR(w,{\sigma }_{u}^{2})$. To overcome the difficulty of choosing between the spatially structured and unstructured effects, one can sum up the two priors, resulting in the so-called *convolution priors* of the two independent components. Thus

4 $$\mathrm{log}\,{\varsigma }_{it}=\alpha +{v}_{i}+{u}_{i}$$

The term ${\upsilon }_{i}+{u}_{i}$ can be interpreted as a random spatial adjustment to the overall intercept, and $\alpha +{\upsilon }_{i}+{u}_{i}$ as varying intercepts across areas. To account for time and varying space-time effects, further parameterization of the model results in

5 $$\mathrm{log}\,{\varsigma }_{it}=\alpha +{v}_{i}+{u}_{i}+{\rho }_{t}+{\phi }_{it}$$

where $\rho$ accounts of the temporal processes and ${\phi }_{it}=\{{\phi }_{11},\mathrm{...},{\phi }_{nT}\}$ are space-time random interactions. Considering the years $t=1,\mathrm{...},T$ as Gaussian vectors, one can specify either first or second-order random walk processes for ${\rho }_{t}$. First and second-order random walk processes are specified as ${\rho }_{t}={\rho }_{t-1}+{\rm{\Delta }}\rho$ and ${\rho }_{t}=2{\rho }_{t-1}-{\rho }_{t-2}+{\rm{\Delta }}\rho$, receptively, where ${\rm{\Delta }}\rho  \sim N(0,{\sigma }_{\rho }^{2})$. The first-order random walk penalizes abrupt jumps, while a second-order random walk penalizes deviations from the linear trend $2{\rho }_{t-1}-{\rho }_{t-2}$. The space-time interaction effects ${\phi }_{it}$ may be specified either of the four ways: unstructured temporal and unstructured spatial effects, structured temporal and unstructured spatial effects, unstructured temporal and structured spatial effects, or structured temporal and structured spatial effects. Details of theses specifications can be found in Knorr-Held^25^.

The model can further be adjusted to accommodate the joint effects of area-level continuous covariates ${x}_{ip}$,$p=1,\mathrm{...},P$, and categorical covariates ${z}_{ik}$ $k=1,\mathrm{...},K$

6 $$\mathrm{log}\,{\varsigma }_{it}=\alpha +{v}_{i}+{u}_{i}+\rho t+{\phi }_{it}+{\rm{\Sigma }}{\beta }_{p}{x}_{ip}+{\rm{\Sigma }}{\gamma }_{k}{z}_{ik}$$

where ${x}_{ip}$ is the *p*th continuous regressor at location *i* with coefficients ${\beta }_{p}$, and ${z}_{ik}$ is the is the *k*th categorical regressor at location *i* with coefficients ${\gamma }_{k}$.

The model can be reparameterized to accommodate spatial variability of the covariates effects by varying the coefficients across areas. A spatially varying coefficient version is

7 $$\mathrm{log}\,{\varsigma }_{it}=\alpha +{v}_{i}+{u}_{i}+\rho t+{\phi }_{it}+{\rm{\Sigma }}({\beta }_{p}+{\delta }_{ip}){x}_{ip}+{\rm{\Sigma }}({\gamma }_{k}+{\delta }_{ik}){z}_{ik}$$

where ${\delta }_{ip}$ and ${\delta }_{ik}$ are differential spatially varying effects which account for varying effects of the covariates. Thus, ${\beta }_{ip}={\beta }_{p}+{\delta }_{ip}$ and ${\gamma }_{ik}={\gamma }_{k}+{\delta }_{ik}$ can be viewed as random slope processes. The common specifications for ${\delta }_{i}=\{{\delta }_{ip},{\delta }_{ik}\}$ are either ICAR processes ${\delta }_{i} \sim ICAR(w,{\sigma }_{\delta }^{2})$ or exchangeable Gaussian processes ${\delta }_{i} \sim N(0,{\sigma }_{\delta }^{2})$.

### Bayesian Inference

Let ${{\boldsymbol{\psi }}}_{{\bf{1}}}{\boldsymbol{=}}\{{\boldsymbol{\varsigma }},{\boldsymbol{\alpha }}{\boldsymbol{,}}{\boldsymbol{\beta }}{\boldsymbol{,}}{\boldsymbol{\gamma }},{\boldsymbol{\delta }}{\boldsymbol{,}}{\bf{v}}{\boldsymbol{,}}{\bf{u}}{\boldsymbol{,}}{\boldsymbol{\rho }}{\boldsymbol{,}}{\boldsymbol{\phi }}\}$ be the full Gaussian latent (unobservable) field and ${{\boldsymbol{\psi }}}_{{\bf{2}}}=\{{\sigma }_{\delta }^{2},{\sigma }_{v}^{2},{\sigma }_{u}^{2},{\sigma }_{\rho }^{2},{\sigma }_{\phi }^{2}\}$ be a vector of hyper-parameters. The components of ${{\boldsymbol{\psi }}}_{{\bf{1}}}$ are conditionally independent with the sparse precision matrix ${{\rm{\Omega }}}_{ij}=0$ for $i\ne j$. Hence we assume a multivariate normal prior ${{\boldsymbol{\psi }}}_{1} \sim MVN(0,{{\boldsymbol{\Omega }}}^{{\boldsymbol{-}}1})$ with conditional density

8 $$p({{\boldsymbol{\psi }}}_{{\bf{1}}}|{{\boldsymbol{\psi }}}_{{\bf{2}}})={(2\pi )}^{n/2}{|{\boldsymbol{\Omega }}|}^{1/2}\exp (-\frac{1}{2}{{\boldsymbol{\psi }}}_{{\bf{1}}}^{{\bf{T}}}{\boldsymbol{\Omega }}{{\boldsymbol{\psi }}}_{{\bf{1}}})$$

The conditional density is a Gaussian random field of which the sparsity of Ω gives rise to computational benefits. Consequently, we adopted a Bayesian hierarchical specification through the INLA to estimate the model parameters. INLA provides accurate estimates of the integrals through Laplace approximation, a deterministic algorithm proposed by Rue and Martino^21^. By the Bayesian paradigm, we require calculating the posterior distribution of the unknown parameters given the data. Hence, the model parameters are assumed random with prior distributions assigned at each stage of the hierarchy. The modeling can be summarized under a three stage hierarchical framework; the data model, process model and parameter model. Thus:

Stage 1: $${\bf{y}}|{{\boldsymbol{\psi }}}_{{\bf{1}}}{\boldsymbol{,}}{{\boldsymbol{\psi }}}_{{\bf{2}}}{\boldsymbol{ \sim }}p({\bf{y}}|{{\boldsymbol{\psi }}}_{{\bf{1}}}{\boldsymbol{,}}{{\boldsymbol{\psi }}}_{{\bf{2}}})$$

Stage 2: $${{\boldsymbol{\psi }}}_{{\bf{1}}}|{{\boldsymbol{\psi }}}_{{\bf{2}}} \sim p({{\boldsymbol{\psi }}}_{{\bf{1}}}|{{\boldsymbol{\psi }}}_{{\bf{2}}})$$

Stage 3: $${{\boldsymbol{\psi }}}_{{\bf{2}}} \sim p({{\boldsymbol{\psi }}}_{{\bf{2}}})$$

The joint posterior distribution of ${{\boldsymbol{\psi }}}_{1}$ and ${{\boldsymbol{\psi }}}_{2}$ given the data likelihood is

9 $$p({{\boldsymbol{\psi }}}_{1},{{\boldsymbol{\psi }}}_{2}|{\bf{y}})=\frac{p({{\boldsymbol{\psi }}}_{1},{{\boldsymbol{\psi }}}_{2},{\bf{y}})}{p({\bf{y}})}=\frac{p({\bf{y}}|{{\boldsymbol{\psi }}}_{1},{{\boldsymbol{\psi }}}_{2})p({{\boldsymbol{\psi }}}_{1}|{{\boldsymbol{\psi }}}_{2})p({{\boldsymbol{\psi }}}_{2})}{{\int }_{{{\boldsymbol{\psi }}}_{1}}{\int }_{{{\boldsymbol{\psi }}}_{2}}p({\bf{y}}|{{\boldsymbol{\psi }}}_{1},{{\boldsymbol{\psi }}}_{2})p({{\boldsymbol{\psi }}}_{1}|{{\boldsymbol{\psi }}}_{2})p({{\boldsymbol{\psi }}}_{2})d{{\boldsymbol{\psi }}}_{1}d{{\boldsymbol{\psi }}}_{2}}$$

The joint posterior is written in shorthand as $p({{\boldsymbol{\psi }}}_{1},{{\boldsymbol{\psi }}}_{2}|{\bf{y}})\propto p({\bf{y}}|{{\boldsymbol{\psi }}}_{1},{{\boldsymbol{\psi }}}_{2})\times p({{\boldsymbol{\psi }}}_{1},|{{\boldsymbol{\psi }}}_{2})\times p({{\boldsymbol{\psi }}}_{2})$ since the denominator is integrated over the latent field parameters and the hyper-parameters. Using INLA, the integrals are numerically evaluated through nested Laplace approximations. The implementation of INLA can be summarized as follows:

Computation of the joint posterior of the hyper-parameters through nested approximations

10 $$p({{\boldsymbol{\psi }}}_{{\bf{2}}}|{\bf{y}}){\boldsymbol{=}}\frac{p({{\boldsymbol{\psi }}}_{{\bf{1}}}{\boldsymbol{,}}{{\boldsymbol{\psi }}}_{{\bf{2}}}|{\bf{y}})}{p({{\boldsymbol{\psi }}}_{{\bf{1}}}|{{\boldsymbol{\psi }}}_{{\bf{2}}}{\boldsymbol{,}}{\bf{y}})}\approx {\frac{p({\bf{y}}|{{\boldsymbol{\psi }}}_{{\bf{1}}}{\boldsymbol{,}}{{\boldsymbol{\psi }}}_{{\bf{2}}})p({{\boldsymbol{\psi }}}_{{\bf{1}}}|{{\boldsymbol{\psi }}}_{{\bf{2}}})p({{\boldsymbol{\psi }}}_{{\bf{2}}})}{\tilde{p}({{\boldsymbol{\psi }}}_{{\bf{1}}}|{{\boldsymbol{\psi }}}_{{\bf{2}}}{\boldsymbol{,}}{\bf{y}})}|}_{{{\boldsymbol{\psi }}}_{{\bf{1}}}{{\boldsymbol{\psi }}}_{{\bf{2}}}{\boldsymbol{=}}{{\boldsymbol{\psi }}}_{{\bf{2}}}^{{\boldsymbol{\ast }}}({{\boldsymbol{\psi }}}_{{\bf{1}}})}$$

Next, a simplified Laplace approximation approach based on Taylor’s series expansion is used to approximate the posterior marginal $p({{\boldsymbol{\psi }}}_{{\bf{1}}}|{\bf{y}})$. Thus

11 $$\tilde{p}({{\boldsymbol{\psi }}}_{{\bf{1}}}|{\bf{y}})\approx {\frac{p({{\boldsymbol{\psi }}}_{{\bf{1}}}{\boldsymbol{,}}{{\boldsymbol{\psi }}}_{{\bf{2}}}|{\bf{y}})}{\tilde{p}({{\boldsymbol{\psi }}}_{{\boldsymbol{-}}{\bf{i}}1}|{{\boldsymbol{\psi }}}_{{\bf{i}}1}{\boldsymbol{,}}{{\boldsymbol{\psi }}}_{{\bf{2}}}{\boldsymbol{,}}{\bf{y}})}|}_{{{\boldsymbol{\psi }}}_{{\boldsymbol{-}}{\bf{i}}1}{\boldsymbol{=}}{{\boldsymbol{\psi }}}_{{\boldsymbol{-}}{\bf{i}}1}^{{\boldsymbol{\ast }}}({{\boldsymbol{\psi }}}_{{\bf{i}}1}{\boldsymbol{,}}{{\boldsymbol{\psi }}}_{{\bf{2}}})}$$

where $\tilde{p}({{\boldsymbol{\psi }}}_{{\boldsymbol{-}}{\bf{i}}1}|{{\boldsymbol{\psi }}}_{{\bf{i}}1}{\boldsymbol{,}}{{\boldsymbol{\psi }}}_{{\bf{2}}}{\boldsymbol{,}}{\bf{y}})$ is the Laplace-Gaussian approximation to $p({{\boldsymbol{\psi }}}_{{\boldsymbol{-}}{\bf{i}}1}|{{\boldsymbol{\psi }}}_{{\bf{i}}1}{\boldsymbol{,}}{{\boldsymbol{\psi }}}_{{\bf{2}}}{\boldsymbol{,}}{\bf{y}})$ and ${{\boldsymbol{\psi }}}_{{\boldsymbol{-}}{\bf{i}}1}^{{\boldsymbol{\ast }}}({{\boldsymbol{\psi }}}_{{\bf{i}}1}{\boldsymbol{,}}{{\boldsymbol{\psi }}}_{{\bf{2}}})$ is its mode. Lastly, the marginal posteriors are computed as $\tilde{p}({{\boldsymbol{\psi }}}_{1{\bf{i}}}|{\bf{y}})\approx \int \tilde{p}({{\boldsymbol{\psi }}}_{1{\bf{i}}}|{{\boldsymbol{\psi }}}_{{\bf{2}}}{\boldsymbol{,}}{\bf{y}})\tilde{p}({{\boldsymbol{\psi }}}_{{\bf{2}}}|{\bf{y}}){\bf{d}}{{\boldsymbol{\psi }}}_{{\bf{2}}}$.

## Application

### Study area and data

Ghana is centrally located on the west coast of Africa. It has a total land area of 238,589 km^2^ and bordered by Cote d’Ivoire to the west, Togo to the east, Burkina Faso to the north, and the Atlantic Ocean to the south. The country consists of ten administrative regions which are subdivided into 216 districts. Population projection by the Ghana Statistical Service (GSS) at the end of 2014 puts Ghana’s population at 27,043,093. The disease data used for this study consist of yearly diarrhea morbidity records of outpatient departments (OPD) from 2010 to 2014. We obtained the data from the Centre for Health Information and Management (CHIM) of the Ghana Health Services (GHS). These data exist in aggregated format per administrative districts. The geographical scale of analysis was restricted to the 170 administrative districts of which data had been recorded. We obtained population and sociodemographic data from the Ghana Statistical Service (GSS). We constructed four sociodemographic indicators as risk factors of diarrhea. These included *unsafe drinking water* ($uw$), *unsafe toilet* ($ut$), and *unsafe liquid waste disposal* ($ud$), and *urbanization* ($ur$). We estimated $uw$ as the percentage of the district’s population who do not have access to pipe-borne water (either in dwellings, outside dwellings, or public standpipes). We estimated $ut$ as the percentage of the district’s population who do not have access to flush toilet, and $ud$ as the percentage of the district’s population who dispose liquid waste either on the streets or the compound. The variable $ur$ indicates the percentage of the district’s population who live in urban communities. We classified $ur$ as rural (less than 30% urban population), peri-urban (30–70% of urban population) and urban (greater than 70% urban population).

### Model implementation

The case study for model specification involved diarrhea outcomes disaggregated by $i=1,\mathrm{...},170$ districts over $t=1,\mathrm{...},5$ years. We fitted three separate models. We specified ${v}_{i} \sim N(0,{\sigma }_{v}^{2})$ for the unstructured spatial effects, ${u}_{i} \sim ICAR(w,{\sigma }_{u}^{2})$ for the structured spatial effects. For the temporal effects, we specified first-order random walk prior ${\rho }_{t}={\rho }_{t-1}+\Delta \rho$, $\Delta \rho  \sim N(0,{\sigma }_{\rho }^{2})$ because of the short temporal span of the data. Since ${u}_{i}$ and ${\rho }_{t}$ capture the spatially and temporally structured variabilities, we specified ${\phi }_{it} \sim N(0,{\sigma }_{\phi }^{2})$.

**Model 1**: Spatio-temporal fixed effect regression model.

12 $$\begin{array}{c}\mathrm{log}\,{\varsigma }_{it}=\alpha +{v}_{i}+{u}_{i}+{\rho }_{t}+{\phi }_{it}+\\ \quad \quad \quad \,\,\,{\beta }_{uw}uw+{\beta }_{ut}ut+{\beta }_{ud}ud+{\gamma }_{ur}ur\end{array}$$

**Model 2**: This is a spatially varying coefficients model where {*δ*_*uw*,*i*_, *δ*_*ut*,*i*_, *δ*_*ud*,*I*_, *δ*_*ur*,*i*_}$\sim N(0,{\sigma }_{\delta }^{2})$. The premise of this model derives from the assumption that the varying coefficients are Gaussian random processes.

13 $$\begin{array}{c}\mathrm{log}\,{\varsigma }_{it}=\alpha +{v}_{i}+ui+{\rho }_{t}+{\phi }_{it}+({\beta }_{uw}+{\delta }_{uw,i})uw\\ \quad \quad \quad \,\,+({\beta }_{ut}+{\delta }_{ut,i})ut+({\beta }_{ud}+{\delta }_{ud,i})ud\\ \quad \quad \quad \,\,+({\gamma }_{ur}+{\delta }_{ur,i})ur\end{array}$$

**Model 3**: The premise of Model 3 derives from evidence of spatial autocorrelation of the sociodemographic risk factors $uw$, $ut$, $ud$, $ur$. This will suggest ICAR process ${\delta }_{i} \sim ICAR(w,{\sigma }_{\delta }^{2})$ for the covariates with significant spatial autocorrelation. We tested the risk factors for evidence of spatial autocorrelation using the Moran’s index. We then fitted Model 3 as:

14 $$\begin{array}{c}\mathrm{log}\,{\varsigma }_{it}=\alpha +{v}_{i}+{u}_{i}+{\rho }_{t}+{\phi }_{it}+({\beta }_{uw}+{\delta }_{uw,i})wat\\ \quad \quad \quad \,\,+({\beta }_{ut}+{\delta }_{ut,i})toi+({\beta }_{ud}+{\delta }_{ud,i})ud\\ \quad \quad \quad \,\,+({\gamma }_{ur}+{\delta }_{ur,i})ur\end{array}$$

where

$$\{{\delta }_{uw,i},{\delta }_{uw,i},{\delta }_{ud,i},{\delta }_{ur,i}\} \sim \{\begin{array}{c}ICAR(w,{\sigma }_{\delta }^{2})\,if\,Moran\mbox{'}s\,index\,is\,significant\\ N(0,{\sigma }_{\delta }^{2})\quad \quad \quad \,\,Otherwise\end{array}$$

For the prior parameters, we assigned non-informative Gaussian prior distribution with zero mean and precision 10^−5^ for the fixed effects $\{{\beta }_{uw},{\beta }_{ut},{\beta }_{ud},{\gamma }_{ur}\} \sim N(0,{10}^{5})$, and independent diffuse prior for the intercept $p(\alpha )\propto const$. For the precision parameters,${\tau }_{l}=1/{\sigma }_{l}^{2}$, $l=\delta ,u,v,\rho ,\phi$ which are at the lowest level of the hierarchy, the Gamma family are often chosen as conjugate priors. We assigned Gamma distribution of the form $\mathrm{log}\,{\tau }_{l} \sim \,\mathrm{log}\,Gamma(1,0.0005)$.

We compared the predictive performances of the models using the deviance information criterion (DIC) and the mean square error (MSE). We estimated $MSE=0.5{\rm{\Sigma }}{({y}_{it}-{y}_{it}^{\ast })}^{2}$, where ${y}_{it}^{\ast }$ are the predicted counts of diarrhea. The $DIC=\bar{D}+{p}_{D}$ is the sum of the model fit and model complexity^26^. Negative twice the log-likelihood of the deviance ${\bf{D}}({{\boldsymbol{\psi }}}_{{\bf{1}}}|{{\boldsymbol{\psi }}}_{{\bf{2}}})=-2\,\mathrm{log}\,p({\bf{y}}|{{\boldsymbol{\psi }}}_{{\bf{1}}}{\boldsymbol{,}}{{\boldsymbol{\psi }}}_{{\bf{2}}})$ informs the model fit, while the effective number of parameter informs the model complexity. The effective number of parameters,${p}_{D}=\bar{{\bf{D}}}()({{\boldsymbol{\psi }}}_{{\bf{1}}}{\boldsymbol{,}}{{\boldsymbol{\psi }}}_{{\bf{2}}}){\boldsymbol{-}}{\bf{D}}({\bar{{\boldsymbol{\psi }}}}_{{\bf{1}}}{\boldsymbol{,}}{\bar{{\boldsymbol{\psi }}}}_{{\bf{2}}})$, is measured through the difference between the posterior mean deviance $\bar{{\bf{D}}}({{\boldsymbol{\psi }}}_{{\bf{1}}}|{{\boldsymbol{\psi }}}_{{\bf{2}}}){\boldsymbol{=}}{\bf{E}}[{\bf{D}}({{\boldsymbol{\psi }}}_{{\bf{1}}}{\boldsymbol{,}}{{\boldsymbol{\psi }}}_{{\bf{2}}})]$ and the deviance of the posterior mean ${\bf{D}}({\bar{{\boldsymbol{\psi }}}}_{{\bf{1}}}|{\bar{{\boldsymbol{\psi }}}}_{{\bf{2}}}){\boldsymbol{=}}{\bf{D}}({\bf{E}}[({{\boldsymbol{\psi }}}_{{\bf{1}}}{\boldsymbol{,}}{{\boldsymbol{\psi }}}_{{\bf{2}}})])$. Like the MSE, the smaller the DIC value, the better the predictive performance of the model. We fitted all models using the R-INLA package^27^ together with the R software^28^ (R Development Core Team 2016).

## Results and Analysis

### Distribution of covariates

**Figure 1:**
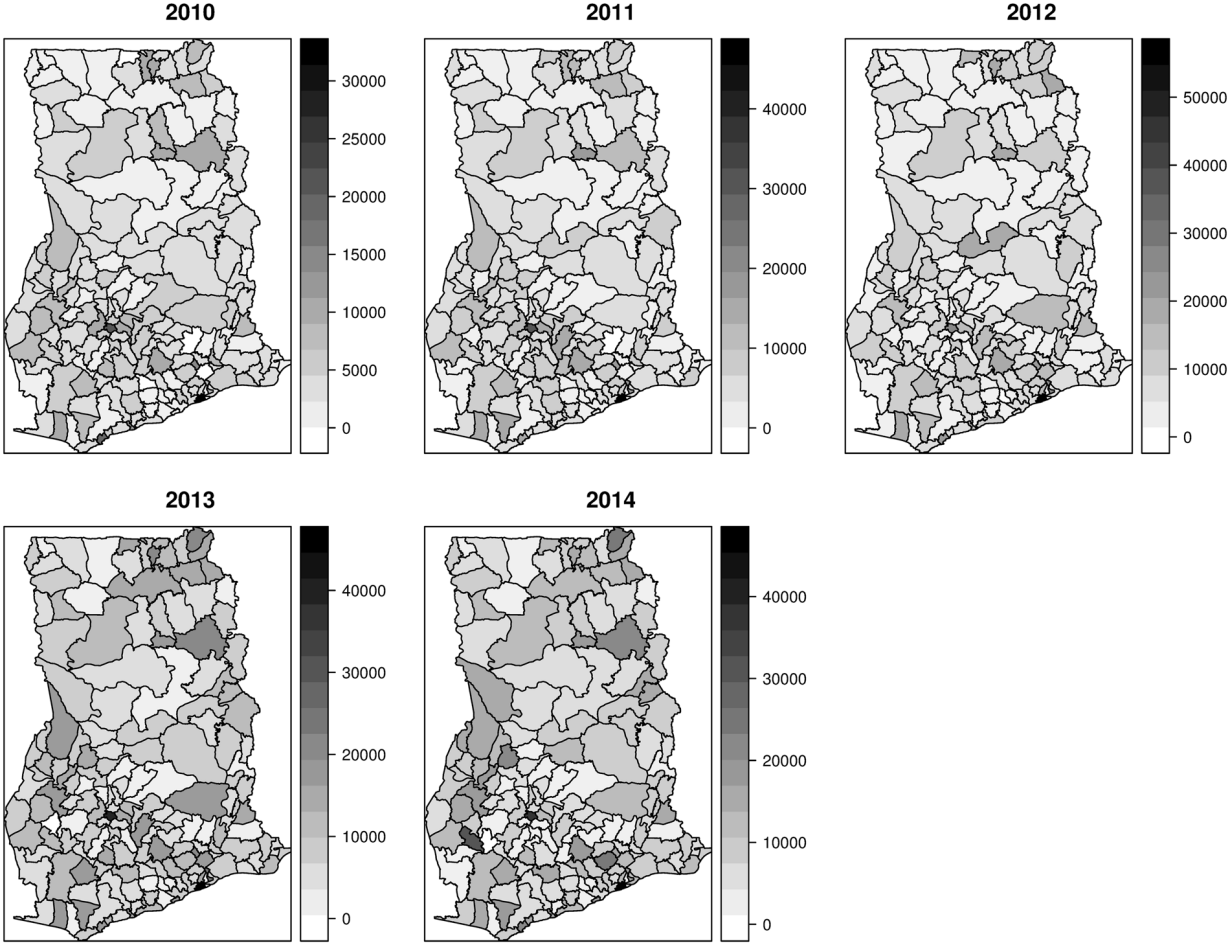
Mapped cases of diarrhea, 2010 to 2014. These maps were created using R software (R Development Core Team 2016)^28^.

**Figure 2:**
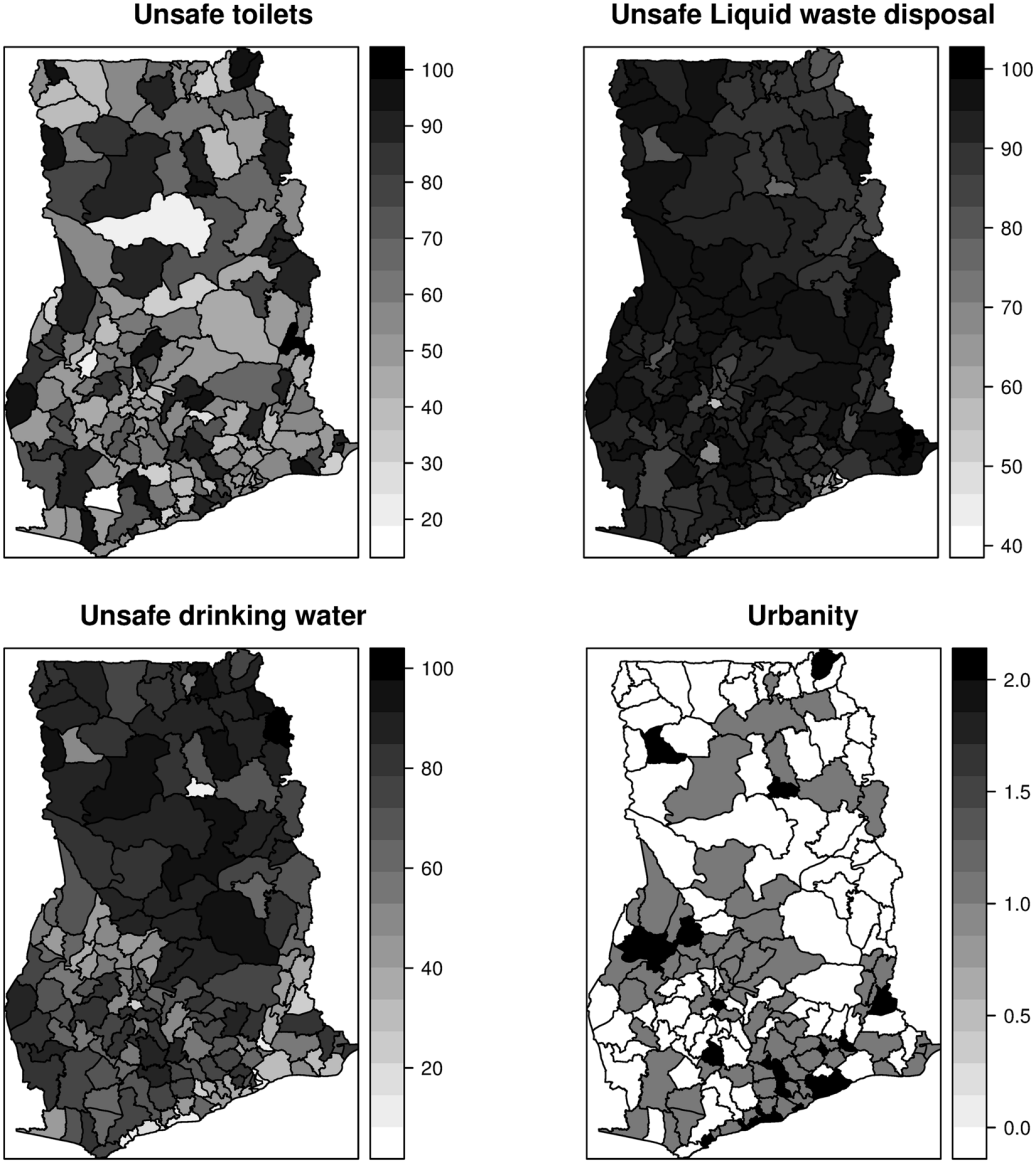
Mapped estimates of the covariates. These maps were created using R software (R Development Core Team 2016)^28^.

Mapped distribution of diarrhea counts from 2010 to 2014 are shown in Fig. 1. Cases ranged from 390 to 3140 in 2010, 590 to 45580 in 2011, 1345 to 54840 in 2012, 357 to 44670 in 2013, and 1069 to 45430 in 2014. Mapped estimates of the covariates are also shown in Fig. 2. The proportion of the population without safe toilets ranged from ≈19% to ≈98%; the proportion without safe drinking sources ranged from ≈8% to ≈98%; the proportion without access to safe liquid waste disposal ranged from ≈42% to ≈99%. Per our classification, 76 out of the 170 districts were dominated by rural dwellers, 67 out of the 170 districts were peri-urban, and urban dwellers dominated 27 out of 170 districts.

### Model selection

**Figure 3:**
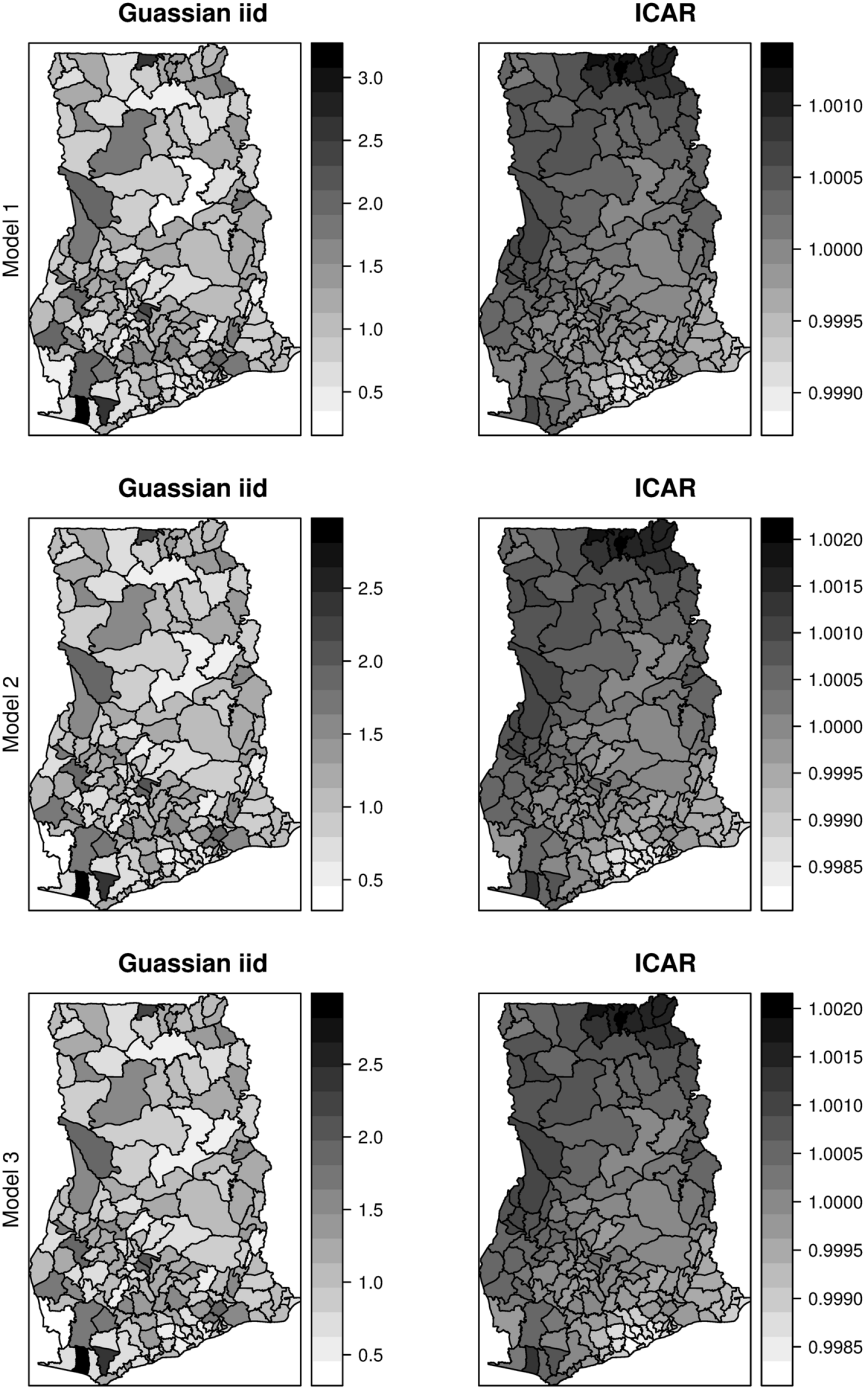
Structured and unstructured spatial effects for Models 1, 2, and 3. These maps were created using R software (R Development Core Team 2016)^28^.

We fitted multiple models and systematically increased the complexity from Model 1 to Model 3. Model 1 is a spatio-temporal regression model with fixed effects. Model 2 and Model 3 rather highlight the spatially varying effects of the sociodemographic factors, an indication of epidemiological benefits. Model 2 is also a spatio-temporal regression model that includes spatially unstructured varying coefficients of the covariates. Moving from Model 1 to Model 2 saw a change in fit as the DIC and the MSE values were reduced from 10601.95 to 10580.32 and from 209.55 to 201.42, respectively (See Table 1). Table 2 shows the results of the test of spatial autocorrelation on the sociodemographic risk factors. We found significant spatial autocorrelation for all the sociodemographic factors except for $ut$. Subsequently, we fitted Model 3 by specifying spatially structured varying coefficients ${\delta }_{i} \sim ICAR(w,{\sigma }_{\delta }^{2})$ for $uw$, $ud$, $ur$, and spatially unstructured varying coefficients ${\delta }_{i} \sim N(0,{\sigma }_{\delta }^{2})$ for $ut$. With the increased complexity, Model 3 adds some improvement regarding fit to Model 2 as the DIC and MSE values were slightly reduced. Both models 2 and 3 highlight a fundamental gain regarding the epidemiological implications on understanding the varying impacts of the covariates. Since the present analysis is concerned with the epidemiological implications of the varying regression coefficients, we focus our further analyses and discussions on Model 3, though the epidemiological implications of Models 2 and 3 will not be different. The relatively lower residual Gaussian random effects of Models 2 and 3 compared with Model 1 (Fig. 3) provide additional support for choosing the varying coefficients models over the fixed effect model.

**Table 1:** Results of the various models fitted.

**Variables**	**Model 1**	**Model 2**	**Model 3**
${e}^{\alpha }$	1.283	1.648	1.648
	(0.249–3.980)	(0.140–7.051)	(0.140–7.051)
***Continuous***			
${e}^{{\beta }_{uw}}$	0.996	0.996	0.996
	(0.991–1.001)	(0.994–1.001)	(0.991–1.001)
${e}^{{\beta }_{ut}}$	0.998	0.998	0.998
	(0.994–1.002)	(0.991–1.001)	(0.993–1.002)
${e}^{{\beta }_{ud}}$	1.117	1.095	1.115
	(0.998–1.257)	(0.961–1.240)	(0.961–1.240)
***Categorical***			
${e}^{{\gamma }_{ur}}$(Rural) (Reference)	1.00	1.00	1.00
${e}^{{\gamma }_{ur}}$(Peri-Urban)	1.129	1.146	1.132
	(0.936–1.347)	(0.953–1.362)	(0.985–1.254)
${e}^{{\gamma }_{ur}}$(Urban)	1.114	1.104	1.108
	(0.773–1.550)	(0.760–1.545)	(0.759–1.547)
*DIC*	10601.95	10580.32	10567.47
*MSE*	209.55	201.42	198.53

**Table 2:** Results of the Moran’s Index of spatial autocorrelation for the varying coefficients in Model 2.

**Variable**	**Moran’s I**	***p*** **-value**
$uw$	0.364	<0.0001
$ut$	−0.092	0.0964
$ud$	0.388	<0.0001
$ur$	0.238	<0.0001

### Distribution of posterior estimates of diarrhea risk

**Figure 4:**
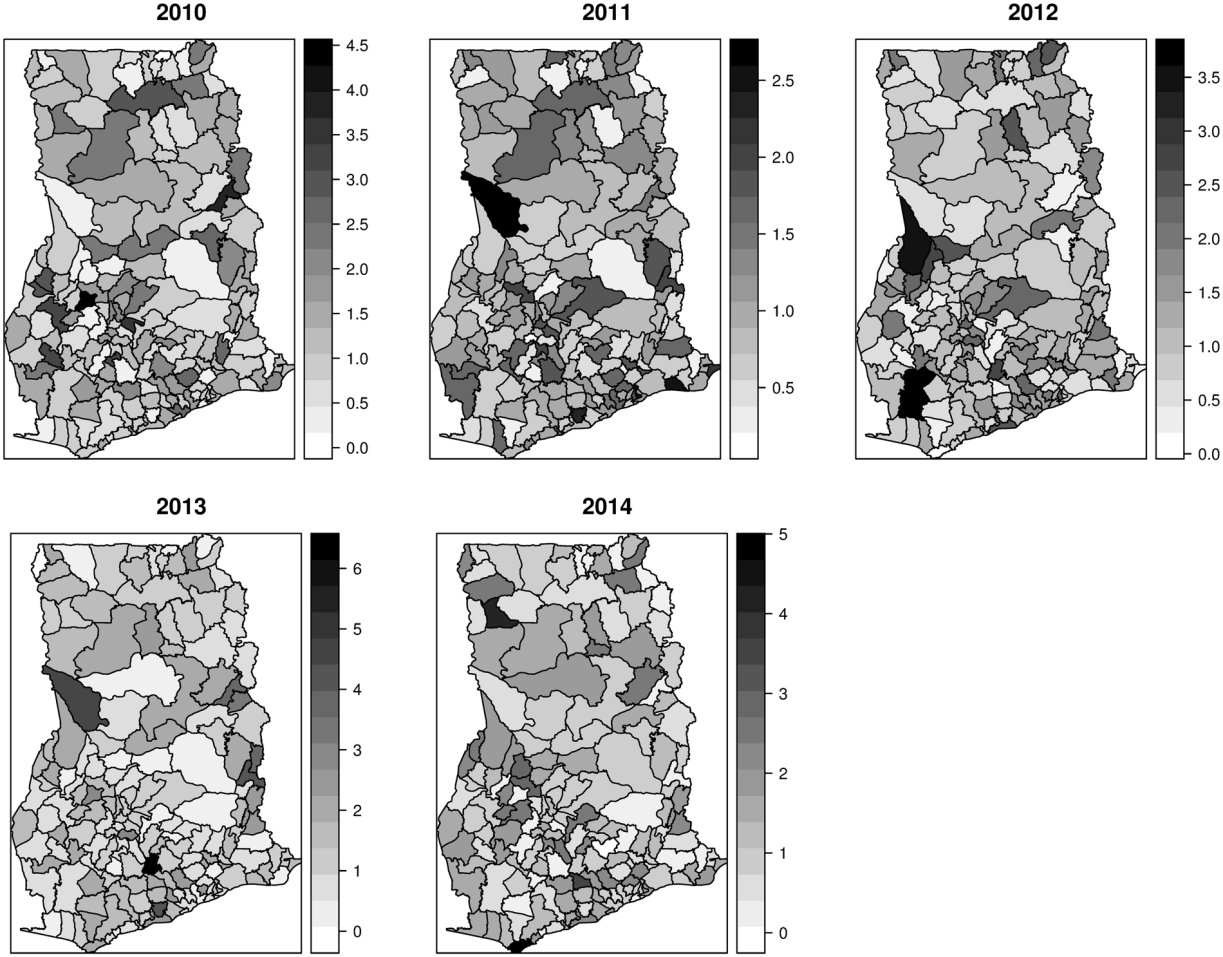
Spatio-temporal distribution of the posterior estimates from 2010 to 2014. These maps were created using R software (R Development Core Team 2016)^28^.

**Figure 5:**
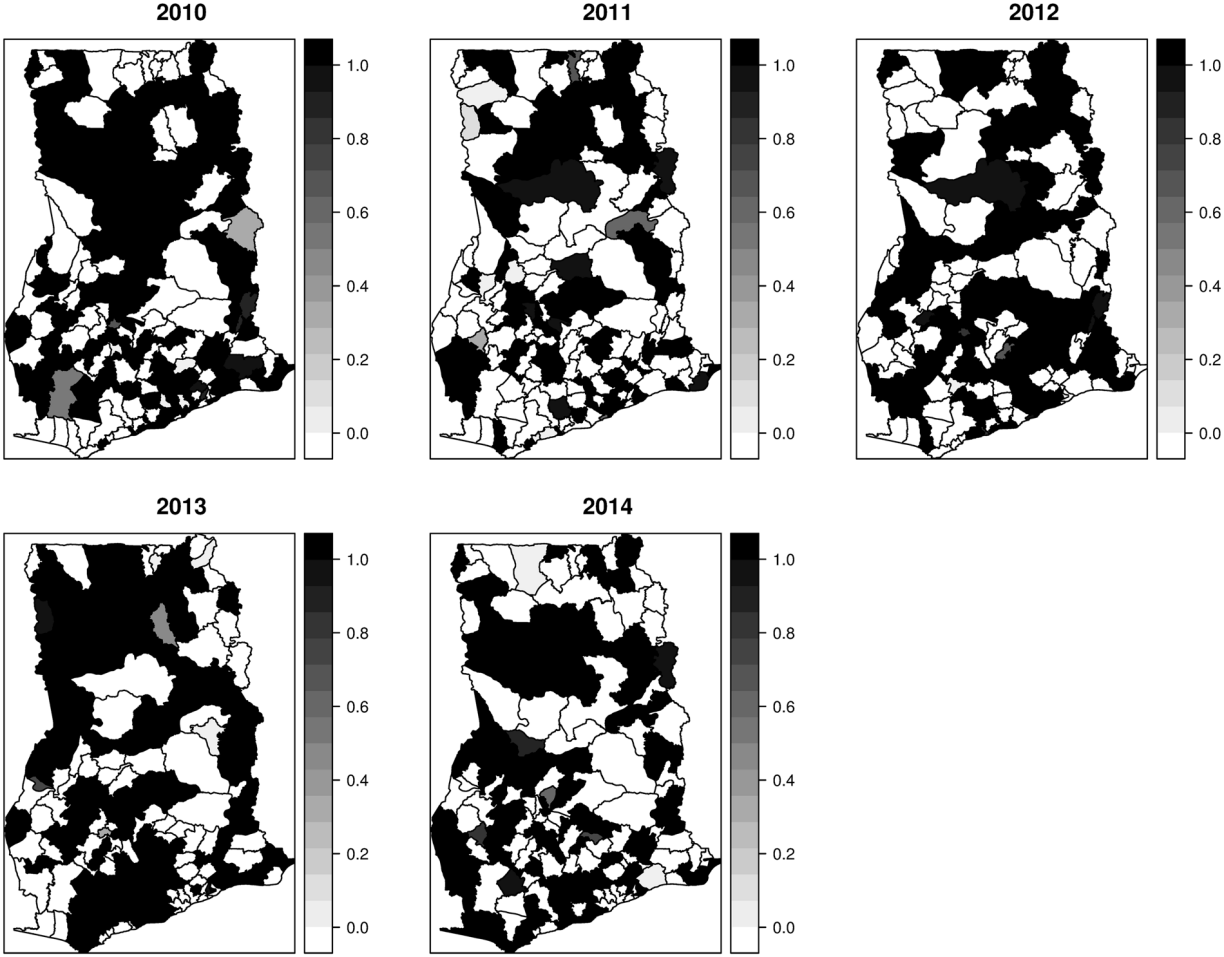
The exceedance probabilities of $p({\varsigma }_{it} > \,1)$, 2010 to 2014. These maps were created using R software (R Development Core Team 2016)^28^.

**Figure 6:**
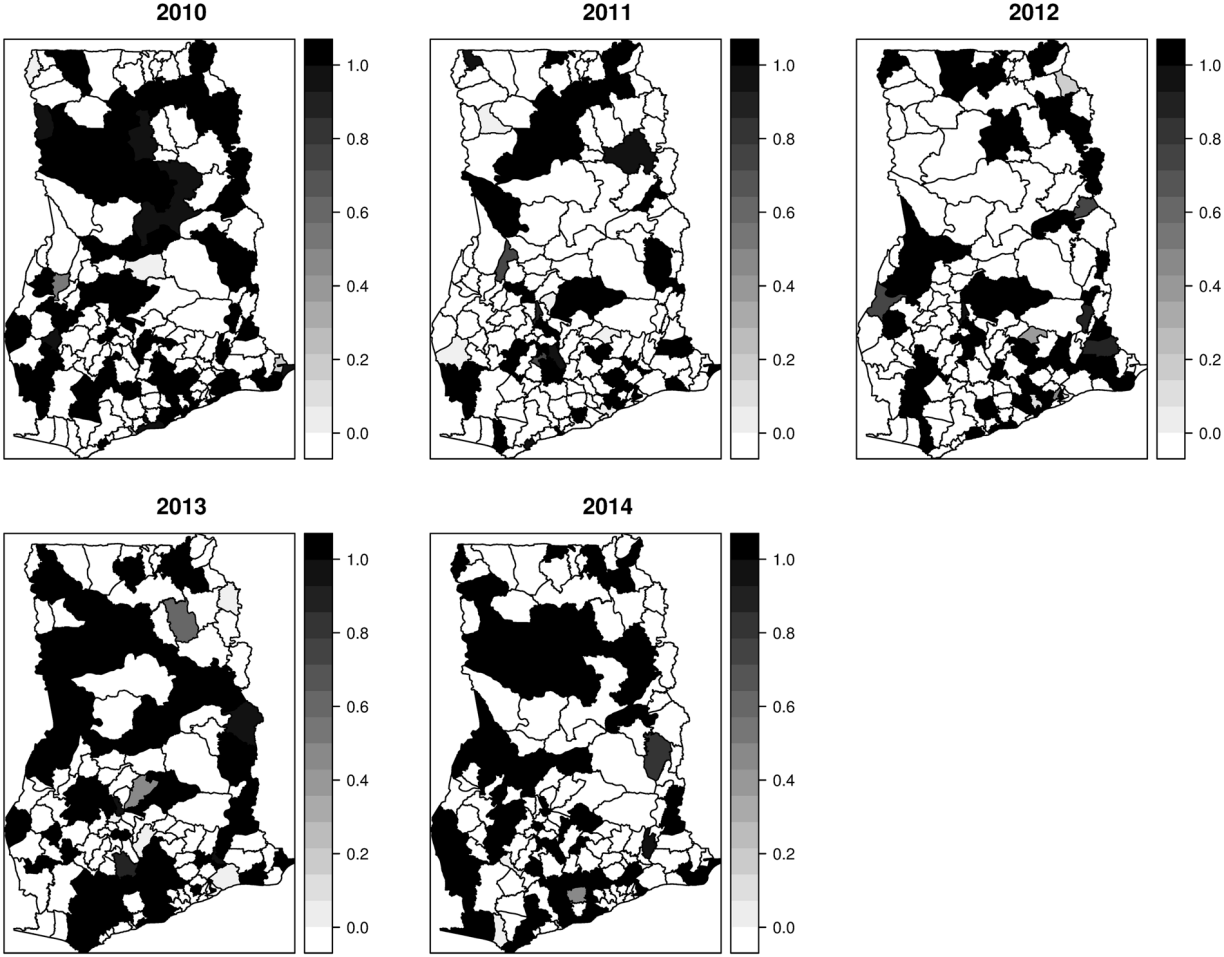
The exceedance probabilities of $p({\varsigma }_{it} > \,1.25)$, 2010 to 2014. These maps were created using R software (R Development Core Team 2016)^28^.

**Figure 7:**
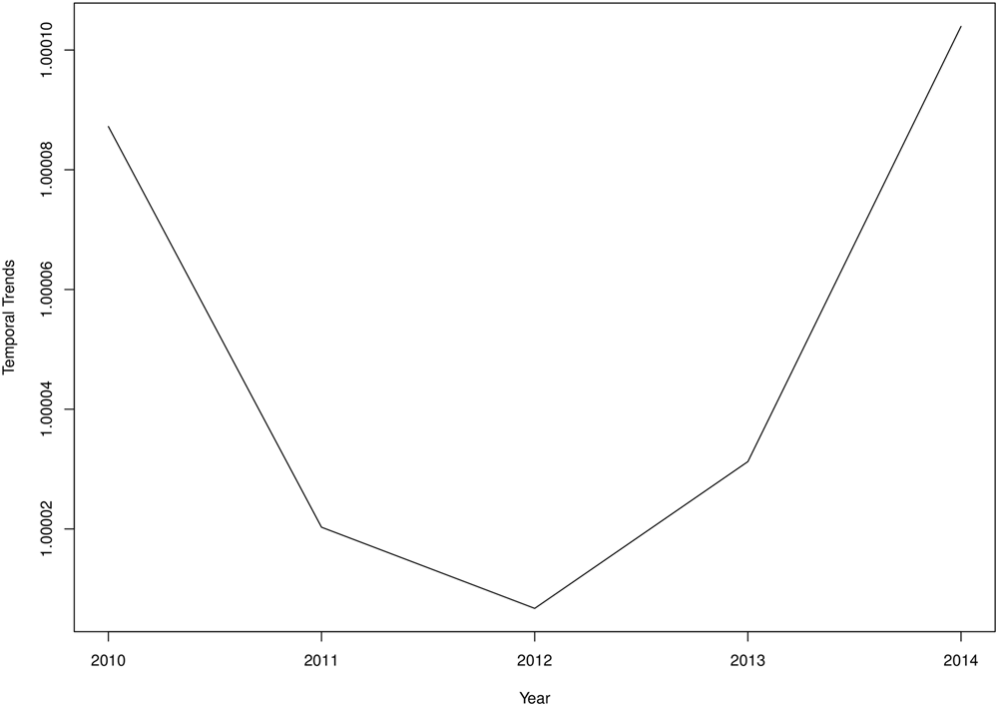
Temporal patterns of diarrhea risk from 2010 to 2014.

All parameters have been transformed on the natural scale to facilitate easy interpretation of their impacts. In this respect, an additive change in the covariates has a multiplicative effect on the risk. Figure 4 shows the spatio-temporal distribution of the posterior estimates of the relative risks ${\varsigma }_{it}$ from 2010 to 2014 after accounting for spatially random and structured effects, space-time interactions, temporal effects, and varying coefficient effects. We interpret these as model-based relative risks. The relative risks considerably contrast across space and appear consistent and notable over time with indistinguishable temporal trends. Areas with ${\varsigma }_{it} > 1$ have higher than expected risk, while those with ${\varsigma }_{it} < 1$ have lower than expected risk. We observed few isolated instances of exceptionally high risk and low risk which appear to emerge and disappear over time. The corresponding exceedance probabilities, $p({\varsigma }_{it} > 1)$ and $p({\varsigma }_{it} > 1.25)$, are shown in Figs 5 and 6, respectively. The darker colors show areas of high probabilities, while the white colors show areas of low probabilities. Supported by the exceedance probability maps, patterns of districts with relative risk ${\varsigma }_{it} > 1$ or ${\varsigma }_{it} > 1.25$ seem spatially continuous. Increasing the exceedance from 1 to 1.25 still showed a majority of the districts with 25% higher than expected risks, except for 2011 and 2012 which had fewer districts. This is in coherence with the temporal patterns which showed a sharp decline in the risk from 2010 to 2012 and increased again from 2012 to 2014 (Fig. 7).

### Structured, unstructured, and temporal effects

**Figure 8:**
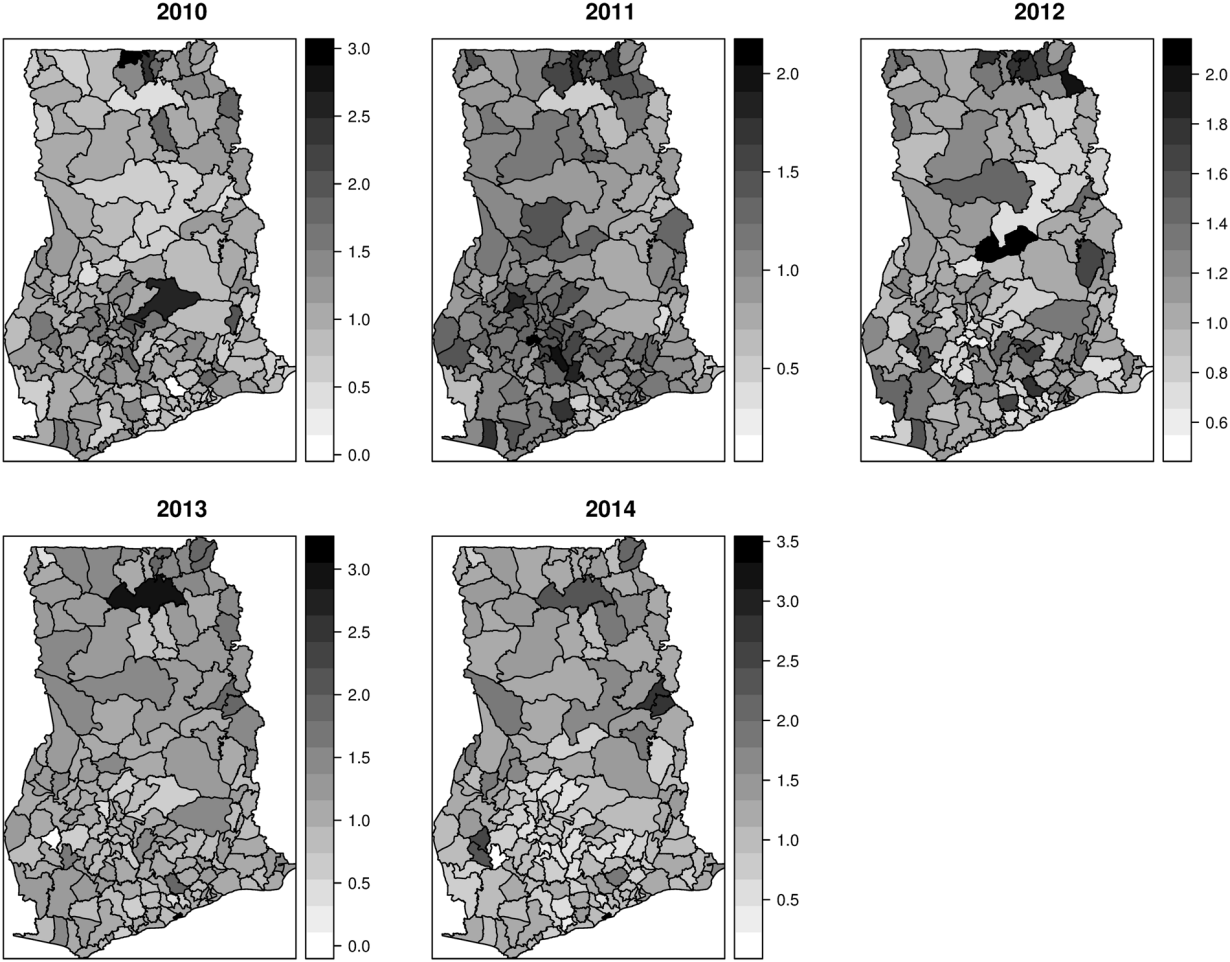
Space-time interaction terms. These maps were created using R software (R Development Core Team 2016)^28^.

The proportion of variance explained by *u*_*i*_ out of the total spatial random effects is ≈1%, suggesting that unstructured heterogeneity ${v}_{i}$ dominates the spatial variability. Additionally, maps of the residual spatial effects indicate unstructured heterogeneity outweighed the structured spatial effects (Fig. 3). The plots of the space-time interaction terms are shown in Fig. 8. These are viewed as residual effects after the spatially structured and unstructured, temporal, and covariates effects have been accounted for in the model. We observed notable spatial patterns as areas of similar values cluster. The temporal patterns appear random, which is in agreement with the modeling assumption. Districts with elevated estimates indicate sporadic or short-term increases in the risk. This appears common amongst many districts in the study area, indicating signs of unstable or unusual temporal trend.

### Spatially varying effects of sociodemographic factors

**Figure 9:**
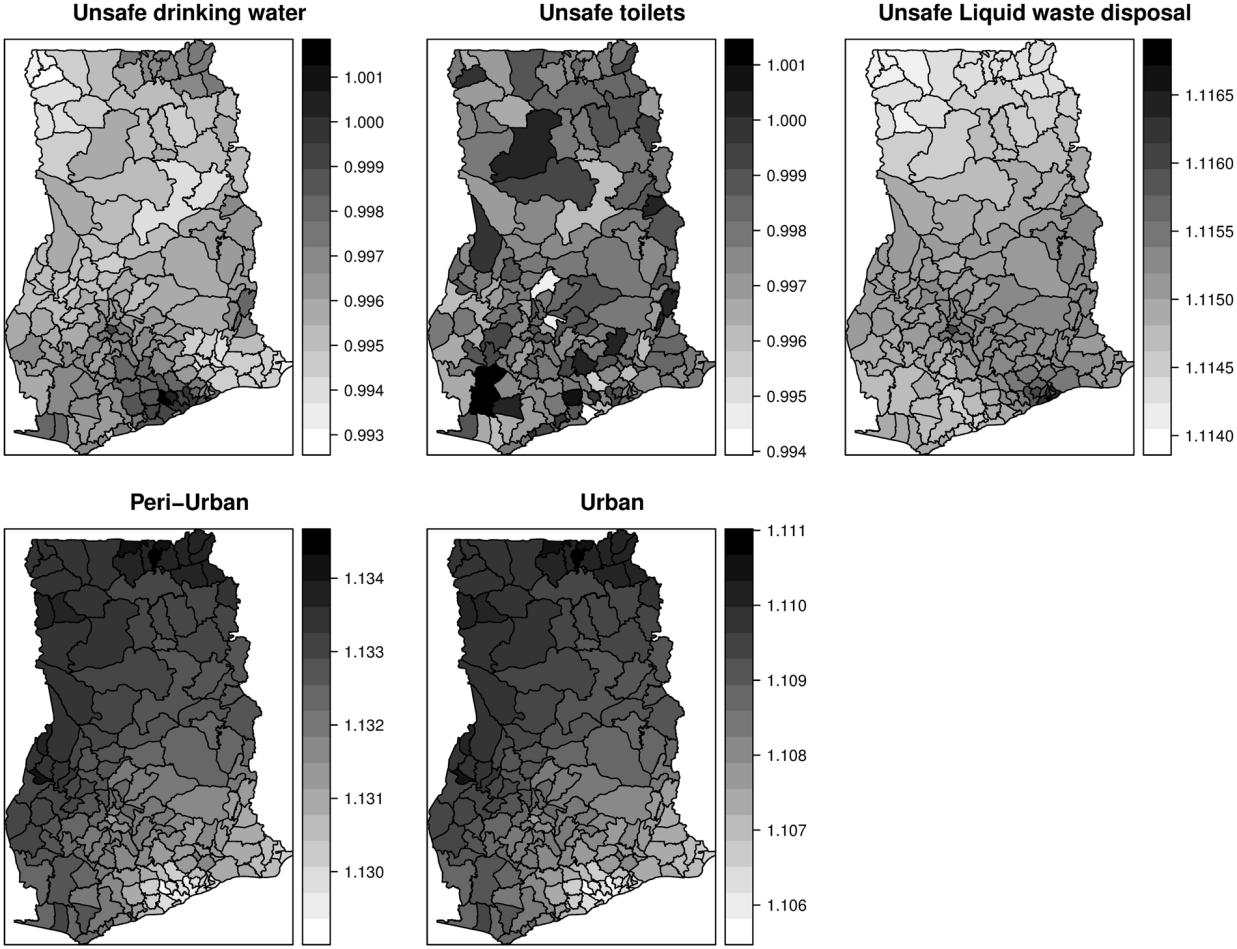
Spatially varying effects of sociodemographic factors. These maps were created using R software (R Development Core Team 2016)^28^.

We observed that a unit increase in the proportion of people with unsafe liquid waste disposal increases diarrhea risk by 11.5%. This predisposes that diarrhea risk for inhabitants with unsafe liquid waste disposal is 11.5% higher than those with safe liquid waste disposal. The risk of diarrhea on average is 10.8% higher in urban (${\gamma }_{ur}=1.108$) and 13.2% higher in peri-urban (${\gamma }_{ur}=1.132$) districts than rural areas districts. The coefficients, however, varied marginally amongst districts (Fig. 9). For peri-urban districts, relatively higher coefficients occurred within northern and south-western parts, while low and no coefficients occurred within the south-eastern parts. The risk in urban districts relative to rural districts ranged varied geographically. We observed lower effects dominating within the southern parts and higher effects within the western parts (Fig. 9).

The multiplicative effect of $dis$ was observed to be ${e}^{{\beta }_{dis}}=1.115$, suggesting that diarrhea risk for inhabitants with unsafe liquid waste disposal is 11.5% higher than those with safe liquid waste disposal. The spatially varying coefficient model rather highlighted variation in these effects (Fig. 9). Higher effects were observed within the south-central parts through to the south-western parts. We observed no multiplicative effect for $uw$, ${e}^{{\beta }_{uw}}\approx 1$ and that of $ut$, ${e}^{{\beta }_{ut}}\approx 1$. This suggests that differences in sources of drinking water and types of toilets could not account for variation in diarrhea risk. The varying coefficients ${e}^{{\beta }_{uw,i}}\approx 1$ and ${e}^{{\beta }_{ut,i}}\approx 1$ similarly showed no multiplicative effects across districts.

## Discussion

This study presented and illustrated the application of a Bayesian hierarchical varying coefficient model to study the sociodemographic effects on diarrhea morbidities, and developed model-based maps of the relative risks. We accounted for local variations in neighborhood covariates effects through spatially varying coefficients. The overriding consequence of our findings is that sociodemographic factors are spatially continuous, hence fixed effects models are inadequate to quantify covariate impacts. Neighborhood morbidities are often aggregated over discrete administrative units such as regions, districts, or census tracts and do not reasonably match the distribution processes of the disease. Disease outcomes misreported across neighborhood boundaries can consequently lead to spatial spillovers. Population heterogeneities also cause variance instability in risk estimates. Adopting spatially structured and unstructured hierarchical random effects model which pools strength over neighborhoods using ICAR and exchangeable intercepts account for the mentioned confounders. An intuitively convenient alternative is a hierarchical Poisson-Gamma model where hyper-priors are assigned to the gamma parameters through the hierarchy ${\varsigma }_{it}|a,b \sim G(a,b)$, with $a|\omega  \sim {h}_{a}(\omega )$, $b|\omega  \sim {h}_{b}(\omega )$, where ${h}_{a}(\cdot )$ and ${h}_{b}(\cdot )$ are the hyper-priors distributions for *a* and *b*, respectively. The major disadvantage of the Poisson-Gamma model lies in its inability to be easily generalized to cope with spatial correlation and covariate adjustment^29^.

Literature on models which seek to account for varying covariate effects on dependence variables remains scarce, especially in epidemiological applications where non-Gaussian outcomes are common. A pioneering methodology is Fotheringham *et al*.’s^13^ GWR which estimates varying coefficients by fitting *n* repeated regression models for separate likelihoods. The departure of our study is that we considered the relative risk as a latent random variable within a single likelihood, and adopted a random effects approach to accommodate all possible confounders. This approach is easily extendable to jointly accommodate independent Gaussian and Gaussian Markov random processes, temporal, and spatio-temporal processes.

Our empirical study indicates evidence of modeling benefit of the spatially varying coefficient models over the fixed effect model regarding fit and epidemiological significance. The relatively large number of districts in our study, *n* = 170, adds to the advantage of adding varying coefficients. The results suggest spatially varying effects of sociodemographic factors on diarrhea risk. There was minimal contrast in the varying coefficients, especially for the continuous covariates, probably because the sociodemographic data were based on population samples. Even though the improvement in fit and variations in the effects seem marginal, the epidemiological advantage of fitting a varying coefficient model cannot be overlooked. Thus, the varying coefficient models can provide added advantage in terms of understanding the substantive epidemiological implications of the varying effects of the sociodemographic factors. We argue that estimates of the varying effects could have revolutionary implications for guiding and prioritizing interventions.

The area specific relative risk maps can be interpreted as model-based relative risks (Fig. 4). These maps suggest considerable contrast across space and appear consistent and notable over time with indistinguishable temporal trends. This may be explained by the varying effects of sociodemographic factors. Site-specific interventions need to consider the relative importance of targeted multiple transmission routes^8^. The model-based risk maps suggest the importance of specific sociodemographic factors at specific locations necessary for reducing morbidities. We observed spatially varying associations between diarrhea risk and sociodemographic factors. Specifically, we found evidence of peri-urban and urban disadvantage as the risk in peri-urban and urban districts were higher than the risk in rural districts. Peri-urban areas are mostly transitional zones often neglected by urban planners. Constant pressure by increasing populations from rural population influx coupled with the high cost of housing in urban districts heightens the potential for creating slums and informal settlements in both urban and peri-urban areas. Such settlements are often plagued with poor water and sanitation which are the well-known driving forces of diarrhea^30, 31^. This aspect of rural-urban differences in diarrhea risk has also been indicated by Kumi-Kyereme and Amo-Adjei^32^ for childhood diarrhea in Ghana using Multiple Indicator Cluster Survey (MICS) data.

A percentage increase in the proportion of the population with unsafe liquid waste disposal was observed to increase diarrhea risk by 11.5%. The implication is that increasing access to safe liquid waste disposal could drastically reduce diarrhea morbidities. This finding was expected as improper disposal of liquid waste increases the potential for fecal contamination and increases the soil moisture content in the environment which plays an intermediate role in diarrhea transmission^33^. Some studies have evaluated this role as an important transmission concern for all diarrhea pathogens, especially in children^34, 35^. Mika *et al*.^36^ noted that with adequate moisture content, some diarrhea-causing fecal indicator bacteria diarrhea decays less rapidly in soil. The varying coefficients showed relatively lower coefficients for this effect for districts within the northern parts. This is probably because the northern parts are dominated by Guinea and Sudan Savannah ecological zones which have much drier soil and high temperature, and likely provide unfavorable environmental and ecological conditions for the survival of diarrhea-causing pathogens. In fact, high temperature has been shown to have a reduced effect on diarrhea^37^.

The observance of similar diarrhea risk amongst districts with safe and unsafe drinking water does not follow the predictable pattern. In the same manner, a recent study in Ghana observed that the incidence of diarrhea was not significantly associated with the quality of household water^32^. The attributable reason could be due to practices associated with fetching, storage, and handling which could contaminate even safe sources of water^38^. In Ghana, the availability of safe water sources in urban populations does not guarantee constant safe water availability due to intermittent supply prompted by high demands. Such urban dwellers, though have access to safe water, constantly resort to alternative water sources such as tanker-supply, and streams and rivers which may have higher levels of bacterial and viral counts. These alternative water sources can easily compromise the immune system due to their sporadic usage, increasing the susceptibility to infection. On the other hand, these alternative water sources for urban and peri-urban dwellers are mostly the main water sources for most rural dwellers who might have, due to their continuous usage, developed immunity. Comparable to differences in drinking water, differences in toilets also had no effect on diarrhea risk. The plausibility of this finding might be explained by the fact that flush toilet, which is considered as the safest, is most common amongst urban dwellers. It is, however, the worse in terms of sanitation when water for flushing is unavailable. Intermittent supply of piped water amongst urban dwellers with flush toilet may thus increase diarrhea risk. The implication is that the provision of safe water sources may not profoundly reduce diarrhea unless other mediating factors such as ensuring a constant supply of the safe water and household sanitation are taken into consideration.

Our study diverges from other studies both in method and scope. Previous diarrhea related studies in Ghana and elsewhere have predominantly focused either on single geographic units or the characteristics of the affected individuals^38–42^. Unlike the prior studies, our study explains the spatial patterns and the varying effects of sociodemographic factors on diarrhea. In addition to the methodological significance, our study also highlights several noticeable epidemiological findings. While urbanization is an advantage to reducing water-borne diseases, its implication in developing countries can be daunting if not driven by the necessary requirements, such as availability of continuous safe water supply and access to safe toilet facilities. Although diarrhea risk was found to be high amongst peri-urban and urban districts than rural districts, these relativities varied in space. We observed comparable diarrhea risks amongst districts with safe and unsafe drinking water, indicating that accessibility does not necessarily mean availability. We, however, recognize that these results could be attenuated if all theoretically relevant variables were included. For instance, some studies have indicated that the poor in urban areas are advertently or inadvertently pushed to marginal areas where environmental health conditions are unsuitable for health^30, 31^. Such unsuitable areas are often associated with the creation of urban slums which lack safe water supply and sanitation which potentially combine to increase the risk of diarrhea. Further studies with interaction effects of urbanization and locality type (slums or non-slums) could demonstrate additional reasons for these findings.

Some limitations of this study deserve to be mentioned. First, this study relied on diarrhea data aggregated over districts level spatial units. Hence individual-level inferences would be inappropriate. Thus our study makes the implicit assumption of homogenous population and morbidity distribution within the districts which could lead to misspecification due to ecological bias^43^. Further studies using rigorous statistical estimations such as the log-Gaussian Cox processes^44^ would be used in further studies to attenuate any possible misspecification. Secondly, the sociodemographic covariates were secondary data based on random sampling of individuals in the districts. Sampling size biases and the probability distribution of sampling outcomes of the covariates have not been accounted for in this study. Thirdly, only sociodemographic risk factors were included in this study, while environmental and climatic factors such as temperature, rainfall, and land use/land cover characteristics which might have an important impact on diarrhea have been absent. We have also not considered possible temporal changes in the sociodemographic risk factors due to data unavailability. Nonetheless, we believe this will have minimal effects on the results since most of the variability was captured by the spatial, temporal, and space-time parameters. Our future studies seek to accommodate some of these limitations, especially augmenting sociodemographic risk factors with environmental factors extracted from remotely sensed images.

## Conclusion

Our study represents a contribution to spatial epidemiology literature by demonstrating both the methodological and epidemiological benefits of spatially varying coefficient modeling in estimating diarrhea risk. In contrast to the fixed effect model, the spatially varying coefficient model provided an added advantage to highlight varying effects of the sociodemographic risk factors. This has a practical implication of providing a scientific basis to facilitate precise targeting of site-specific risk factors for intervention. Our study disclosed the spatially varying nature of the relationship between diarrhea morbidities and urbanization, unsafe liquid waste disposal, unsafe toilets, and unsafe drinking water. The consequence of our results indicates that increased access to safe liquid waste disposal could reduce diarrhea drastically. Moreover, the results suggest that the provision of safe water and toilets will not assure reductions in diarrhea morbidities without ensuring a constant supply of the water. The maps of relative risk and the nature of the relationships provide empirical basis useful for guiding neighborhood health planning and resources allocation. Additionally, the study has provided a framework for health practitioners to (1) estimate, and map model-based area specific relative risk of diarrhea, (2) understand the relation between the relative risk and important sociodemographic factors. Further studies, identifying the spatially varying effects of climatic factors on diarrhea will be worthwhile. Moreover, another question worth further investigation is the sensitivity of the spatially varying intercepts and coefficients to complex definitions of proximity.
